# Non-Destructive Detection of Bone Fragments Embedded in Meat Using Hyperspectral Reflectance Imaging Technique

**DOI:** 10.3390/s20144038

**Published:** 2020-07-21

**Authors:** Jongguk Lim, Ahyeong Lee, Jungsook Kang, Youngwook Seo, Balgeum Kim, Giyoung Kim, Seongmin Kim

**Affiliations:** 1 Rural Development Administration, 310 Nongsaengmyeng-ro, Deokjin-gu, Jeonju 54875, Korea; limjg@korea.kr (J.L.); lay117@korea.kr (A.L.); js72kang@korea.kr (J.K.); yws25@korea.kr (Y.S.); bgkim8732@korea.kr (B.K.); giyoung@korea.kr (G.K.); 2 Department of Bioindustrial Machinery Engineering, College of Agriculture & Life Sciences, Chonbuk National University, 567 Baekje-daero, Deokjin-gu, Jeonju 54896, Korea

**Keywords:** meat, foreign substances, short-wave near infrared, hyperspectral imaging

## Abstract

Meat consumption has shifted from a quantitative to a qualitative growth stage due to improved living standards and economic development. Recently, consumers have paid attention to quality and safety in their decision to purchase meat. However, foreign substances which are not normal food ingredients are unintentionally incorporated into meat. These should be eliminated as a hazard to quality or safety. It is important to find a fast, non-destructive, and accurate detection technique of foreign substance in the meat processing industry. Hyperspectral imaging technology has been regarded as a novel technology capable of providing large-scale imaging and continuous observation information on agricultural products and food. In this study, we considered the feasibility of the short-wave near infrared (SWIR) hyperspectral reflectance imaging technique to detect bone fragments embedded in chicken meat. De-boned chicken breast samples with thicknesses of 3, 6, and 9-mm and 5 bone fragments with lengths of about 20–30-mm are used for this experiment. The reflectance spectra (in the wavelength range from 987 to 1701-nm) of the 5 bone fragments embedded under the chicken breast fillet are collected. Our results suggested that these hyperspectral imaging technique is able to detect bone fragments in chicken breast, particularly with the use of a subtraction image (corresponding to image at 1153.8-nm and 1480.2-nm). Thus, the SWIR hyperspectral reflectance imaging technique can be potentially used to detect foreign substance embedded in meat.

## 1. Introduction

Undoubtedly, meat is a major source of animal proteins in many countries. It is also an excellent source of essential fats, soluble vitamins, and minerals. These important nutrients have specific functions in our body. Meat consumption is gradually increasing because of economic development and improved living standards. Additionally, meat processing companies are launching various new products, and consumers are paying more attention to the quality and safety of meat products. Pork, beef, and poultry meat are the most common types of meat used for these products. Previously, poultry meat was consumed in the form of whole meat. However, the recent consumption trend of poultry meat is changing to meat cuts such as chicken breast, tenderloin, chicken legs, and wings [1]. Among these poultry meat cuts, chicken breast and tenderloin consist of boneless meat (or deboned meat) and are used for preparations such as salads, cold dishes, and seasoned salads. 

With the increase in the consumption of meat cuts and the diversification of processed products, the interest in the quality and safety of meat is also increasing. Therefore, meat processing workers are striving to ensure quality and safety through various processing methods such as cooling [2,3,4], freezing [5], and drying [6,7]. Because of the increase in the consumption of meat cuts, meat processing companies include the removal of foreign substances in the processing line. Foreign substances that are not components of normal foods are unintentionally mixed, thus affecting food safety or quality. Foreign substances can be recognized by the naked eye and can be classified into 3 types—animals, vegetables, and mineral properties. In the case of meat, not only steel and plastic particles but also bone fragments are included in foreign substances. The Food Safety and Inspection Service of the United States Department of Agriculture regulates that meat bone particles greater than 20-mm can cause injury and act as a potential food safety risk based on the procedures of the Public Health Risk Analysis Committee (1995) [8]. Hazards caused by foreign substances are divided into biological, chemical, and physical hazards. In the meat industry, animal (bone fragments, nails, feathers, etc.) and mineral (screw, iron, needles, plastics, etc.) substances can cause physical injury to the consumer’s teeth or mouth. Furthermore, it can lead to legal claims and adversely affect the company’s reputation. In the current meat processing line, bone fragment filtration and removal is manually performed, and accidents have frequently occurred despite many efforts.

To prevent such accidents, many efforts have focused on detecting foreign substances using various engineering techniques. One of the most common techniques for detecting foreign substances in meat is using X-ray images. X-ray inspection is widely applied to the food and agriculture fields and is used to evaluate the quality of agricultural products such as wheat [9], apple [10], and watermelon [11]. It is also used to detect bone fragments, glass, and metal in meat [12,13]. A line scan type of X-ray light source and detector were used to detect bone fragments of chicken meat [14]. However, this X-ray imaging technology has a critical error of up to 40% even in commercially distributed facilities [15]. In addition, obtaining sufficient contrast images when the foreign matter is small or the sample is not homogeneous is difficult. In the case of chicken, non-uniform thickness and various shapes of embedded bone fragments increase the error in acquiring chicken X-ray images [16,17,18]. To compensate for the disadvantage of X-ray examination, some researchers have focused on the real-time and non-destructive detection of bone fragments embedded in chicken meat. These studies included other optical techniques such as nuclear magnetic resonance imaging and ultrasonic detection [19,20].

Among them, the hyperspectral imaging technology (HSI) has been highlighted because it is a non-destructive technique for measuring the quality of agriculture and food fields and for ensuring safety [21,22,23,24]. HIS (also called imaging spectroscopy) is a spectral technology that can acquire both target images including spectral information and the spatial information regarding the measurement object, and it is mainly applied for analysis of chemical images [25]. In accordance with the combination of the light source and hyperspectral camera, hyperspectral imaging systems have enabled the acquisition of spectral information and images in the range from the visible to near infrared wavelengths. Grau [26] focused on the relation between the extent of chicken breast decay and the wavelength range from 400 to 1000-nm. Chicken meat contains more unsaturated fatty acids than other meats; thus, HSI is suitable to image chicken meats to improve quality and safety [15,27,28,29,30].

The aim of this study was to examine the possibility of applying the short-wave near infrared (SWIR) hyperspectral reflectance imaging technique for detecting bone fragments embedded in chicken breast. The specific objectives are as follows:

(1) We obtained reflectance spectra and images of chicken breast using a line scan SWIR hyperspectral system with a wavelength of 987 to 1701-nm.

(2) The possibility of detecting the fragments buried in chicken breast was examined using the difference in images of the two selected wavelengths.

## 2. Materials and Methods

### 2.1. Sample Preparation

**Figure 1:**
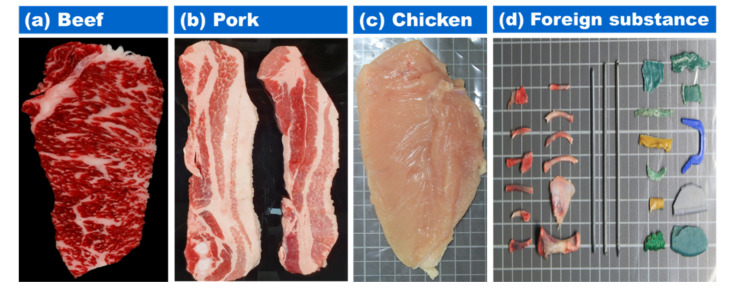
Representative images of meat: (**a**) beef, (**b**) pork, (**c**) chicken breast, and (**d**) foreign substances.

Figure 1 shows beef, pork, and chicken samples which represent sources of protein among meat. The representative animal and mineral foreign matter discovered in the meat processing lines are shown in Figure 1d. Animal foreign substances including bone fragments, hairs, and feathers are left in the washing process, especially bone fragments are not removed completely during the process of bone application in large slaughterhouses or processing plants. Mineral contaminants are often generated in areas where there is a problem with meat processing equipment or where the process control is poor. The types of mineral foreign substances include fragments and plastics.

**Figure 2:**
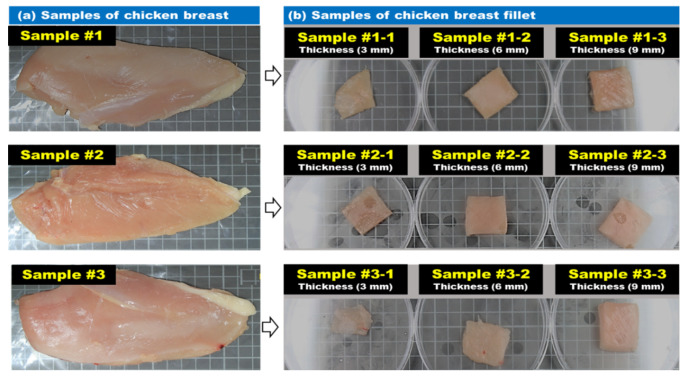
Samples used in the experiment: (**a**) chicken breast meat and (**b**) chicken breast fillet.

The meat used in this study was low in calories, delicate, and soft and had less fat than other meats. Chicken breast (FRESH-UP Gaseumsal, Harim, Iksan, Korea) with the bones removed was used. To facilitate cutting of chicken breast, the samples were stored frozen at −20 °C for 3 h. As shown in Figure 2, frozen chicken breast slices were prepared in 30 × 30-mm squares, and the fillet thicknesses were 3, 6, and 9-mm to obtain reflection spectra and images of chicken breasts of different thickness. A total of nine chicken breast samples were used.

**Figure 3:**
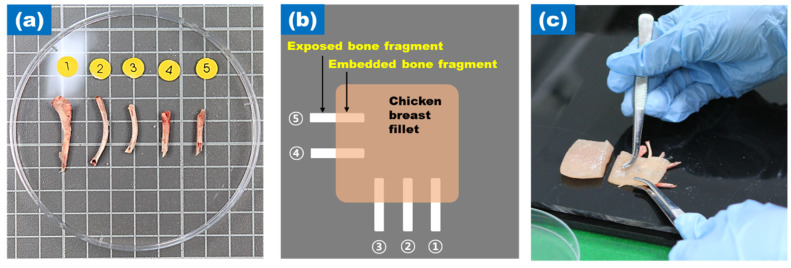
(**a**) 5 bone fragments; (**b**) Schematic diagram of exposed and embedded bone fragments to chicken breast fillet; (**c**) Setting bone fragments to chicken breast fillet.

Bone fragments were manually detected by workers at a poultry processing plant (Harim Corporation, Iksan, Korea), as shown in Figure 3. These bone fragments were utilized to detect targets in the chicken breast, 20 to 30-mm long and 4 to 8-mm wide. The shapes of the bone fragments were not uniform and had sharp features. In Figure 3a, the bone fragments are numbered from 1 to 5 and are arranged as shown in Figure 3b. Half of the bones were placed on the chicken breast fillet, and the other were on the outside to clearly distinguish the bone fragments (Figure 3c).

### 2.2. Push Broom Based Hyperspectral Imaging System

**Figure 4:**
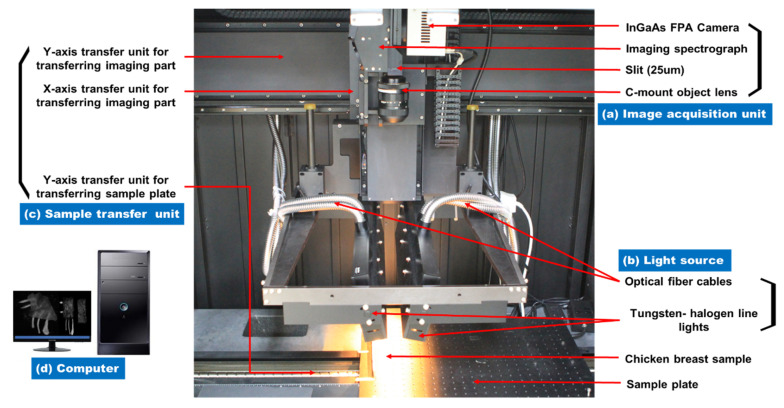
The SWIR hyperspectral reflectance imaging system equipped with (**a**) an image acquisition unit, (**b**) a light source, (**c**) a sample transfer unit, and (**d**) a computer.

A push broom based SWIR hyperspectral reflectance imaging system was established to acquire hyperspectral images of bone fragments embedded in the chicken breast fillet in the reflectance mode, as shown in Figure 4. The system consists of an image acquisition unit (Figure 4a), a light source (Figure 4b), a sample transfer unit (Figure 4c), and a computer (Figure 4d). The image acquisition unit contained an indium gallium arsenide (InGaAs) focal plane array (FPA) camera with a resolution of 640 pixels × 512 pixels (Xeva-3035, Xenics Infrared Solutions, Inc., Leuven, Belgium), an imaging spectrograph (Hyperspec^TM^ NIR G4-249, Headwall Photonics, MA, USA) with a 25-µm slit, and a 35-mm focal length lens (OB-NIR 35/2, Optec, Parabiago, Italy). It was thermo-electrically cooled to −20 °C using a double-stage Peltier device. The line light sources consisted of two 500-mm low-OH fiber optic line lights (LS-F100HS-IR, Seokwang Optical Co. Ltd., Hwasung, Korea) powered by four 150-W tungsten–halogen lamps, optical fiber cables, four DC-stabilized power supplies, and lamp assembly housings. The angle of the two line lights was set to 15° toward the sample. The sample transfer unit included an X-axis and a Y-axis transfer unit for transferring the imaging part and a Y-axis transfer unit for transferring the sample plate. A programmable uniaxial transfer stage (BMS100-UFA, Aerotech, Pittsburgh, PA, USA) moved the sample incrementally across the linear field of view (FOV) of the push broom based hyperspectral imaging system. After passing through a 25-µm (width) × 18-mm (length) aperture slit, light from the FOV scan line was scattered by a scattering grid and projected onto the FPA camera. 2-dimensional images were generated by spatial dimensions along the horizontal axis and spectral dimensions along the vertical axis of the FPA. Image data were digitized at a 14 bit A/D resolution. The system also included a computer for controlling the camera and acquiring hyperspectral images and spectra. All components except the computer were fixed inside a dark chamber to avoid any light, which might affect the veracity of the hyperspectral imaging equipment.

### 2.3. Acquisition of Hyperspectral Reflectance Spectra and Images 

The hyperspectral image contained 3-dimensional data containing one wavelength and 2 spatial dimensions. Each pixel contained a spectrum at a specific location [31]. Table 1 shows the experimental conditions for acquiring SWIR images. The camera exposure time during scanning was set to 20-ms to obtain the SWIR image, the travel distance from one-line scan to the next line scan was set to 0.5-mm using a stepping motor, and the scanning process was performed for a total of 150 times per sample. The wavelength range of the hyperspectral reflectance imaging of the chicken breast fillet was 987–1701-nm (224 bands), and for the images acquired per wavelength, the image resolution was 638 pixels (vertical axis) × 150 pixels (horizontal axis). The data acquired were eventually stored in the computer with the data structure of a 3-dimensional hypercube consisting of 224 bands × 638 pixels × 150 pixels. 

### 2.4. Hyperspectral Image Calibration

The acquired hyperspectral images contained “bad” pixels because of the irregular intensity of the light source and the noise caused by the imaging apparatus. Thus, corrected reflectance images were obtained by applying relative reflectivity, and the bad pixels were removed. To convert the acquired hyperspectral images (R_0_) into the corrected hyperspectral images (R_C_), a white reference image (W) and dark reference image (D) were obtained prior to the measurement. For the white reference image, we used a standard white panel made of Teflon (SRT-151 99-120, Labsphere, New Hampshire, USA) with an optical reflectivity of over 99%, and as the dark reference image, we used the cover of the camera lens to block the light. The obtained raw images were converted into corrected HSI with a total of 224 bands using the following Equation (1):

(1) $$R_{C}=\frac{\left(R_{0}-D\right)}{\left(W-D\right)}$$

### 2.5. Principal Component Analysis 

Principal component analysis (PCA) was performed to extract important information from multivariate data tables and to explain this information using several new variables called principal components (PCs). Calculations are performed repeatedly so that the first PC is the one that carries the most information (or in statistical terms: the most explained variance). The second PC will carry the maximum share of the residual information. PCA is defined as an orthogonal linear transformation. PCs are orthogonal to each other and are ranked to carry more information. PCA is an effective method for identifying patterns in spectra and expressing the spectra in such a way so as to emphasize their similarities and differences. Each spectrum has a score along each principal component. In this study, PCA was used to visualize the hyperspectral reflectance spectra to describe the varieties of samples. 

### 2.6. Image Subtraction Algorithm

An image subtraction algorithm was used to identify small changes between images of the embedded bone fragments and chicken breast fillets. In the hyperspectral imaging process, a mask image (1125-nm band) with the background removed was prepared to remove images other than those of the chicken breast fillets and bone fragments, and a mask was applied to the corrected HSI to obtain the region of interest (ROI) images. As for the operation and image data acquisition of the hyperspectral imaging system, we utilized a program developed using the software development kit provided by the camera manufacturer based on Visual Basic (ver. 6.0, Microsoft, USA). To detect bone fragments covered by the chicken breast fillets, two wavelengths were chosen, and Equation (2) was applied to obtain a subtraction image.

(2) $$S={\mathrm{HSI}}_{\lambda 1}-{\;\mathrm{HSI}}_{\lambda 2}$$

where S is the subtraction image, ${\mathrm{HSI}}_{\lambda 1}$ is the hyperspectral image of band λ1, and ${\mathrm{HSI}}_{\lambda 2}$ is the hyperspectral image of band  λ2. 

## 3. Results and Discussion

### 3.1. Hyperspectral Reflectance Imaging

**Figure 5:**
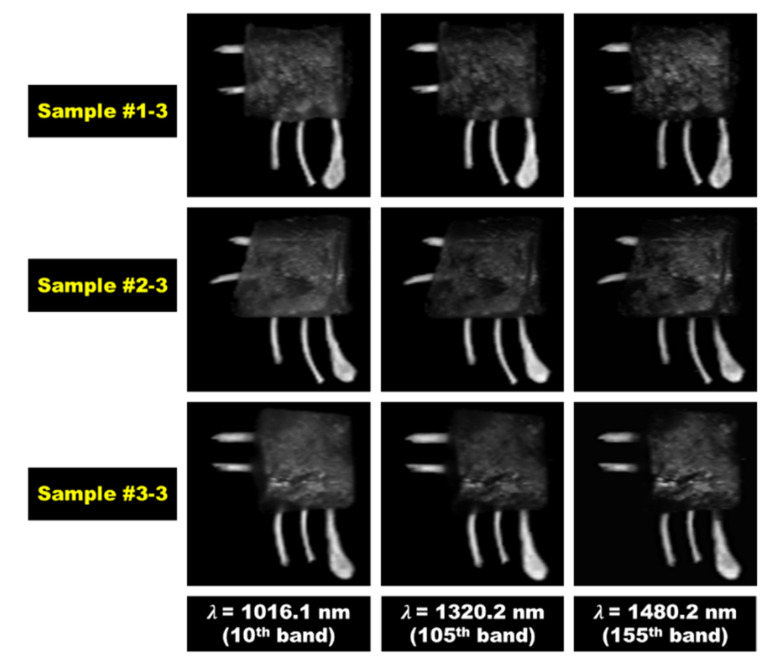
Hyperspectral reflectance images of single wavelength bands obtained from sample #1-3, #2-3, and #3-3 with a thickness of 9-mm.

Figure 5 shows the hyperspectral reflectance images of single wavelength bands obtained from samples 1-3, 2-3, and 3-3 with a thickness of 9-mm. Among the 224 wavelength bands, it was confirmed that the absorption occurred in the chicken breast region in the 1320.2-nm (105th band) and 1480.2-nm (155th band) bands. As shown in Figure 5, the reflectivity of meat to which light was absorbed was weak and close to the black side in the image. In contrast, the bone part looked bright. In this case, the reflectance intensity of the chicken breast fillets was weak, whereas the reflectance intensity of the bone fragments was relatively strong. In the hyperspectral reflectance image corresponding to the single wavelength bands, chicken breast fillets showed very different spectral characteristics compared with the externally exposed bone fragments. However, it was confirmed that bone fragments embedded in the chicken breast fillets were not clearly detected. Hence, we suggested that it will be difficult to detect bone fragments embedded in chicken breast fillets using hyperspectral reflectance images of single wavelength bands.

**Figure 6:**
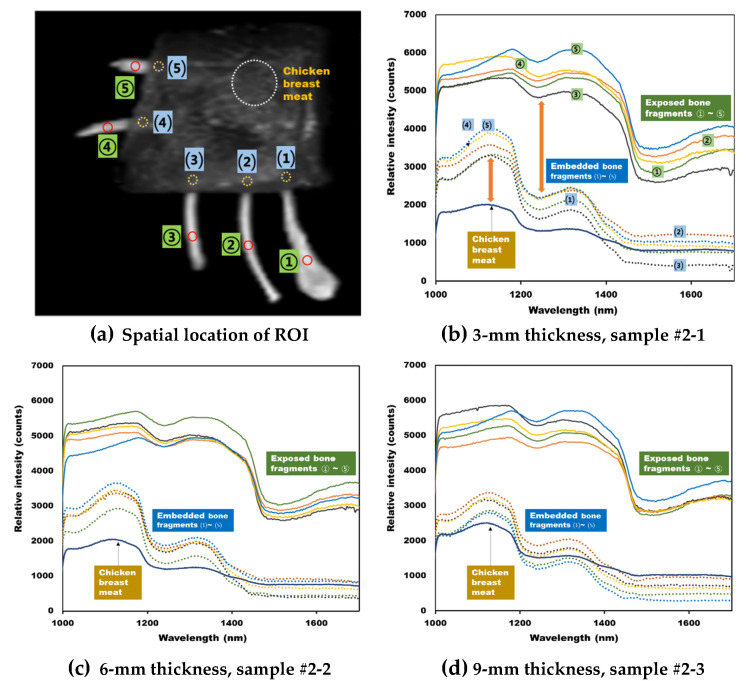
Comparison of the mean spectra extracted from bone fragments and different thicknesses of chicken breast fillets. (**a**) ROI of the hyperspectral image, (**b**) 3-mm thickness, (**c**) 6-mm thickness, and (**d**) 9-mm thickness.

Figure 6a shows the spatial location of ROI for obtaining the average reflectance spectrum for 3 regions (exposed bone fragments, embedded bone fragments, and chicken breast fillet) in sample 2-3. Figure 6b–d show the average spectra for the ROIs at different thicknesses (3-mm, 6-mm, and 9-mm) of chicken breast fillets. In the case of a bone fragments covered with chicken breast fillets, the ROI was obtained by assuming an approximate location of the targets. From the obtained hyperspectral reflectance spectrum, the relative reflectance intensity of the exposed bone fragments was measured to be higher than the built-in bone fragments or pure chicken breast fillets. In the case of bone fragments, the main peaks were observed at wavelengths of 1185 and 1350-nm, and the reflectance sharply decreased at 1535-nm. The wavelengths of 1160 and 1336-nm were the main spectral peaks for the 5 bone fragments covered with chicken breast fillets. As the thickness of the chicken breast fillets increased, the reflection intensity decreased, and the 1336-nm peak drastically decreased. For the portion of bone fragments covered with chicken breast fillets, we assumed that the resulting reflection spectrum included information regarding both chicken breast fillets and bone fragments part. To detect bone fragments embedded in the chicken breast fillet, the relative intensity information of the hyperspectral reflectance spectrum can be used. However, when the thickness of breast fillets was 9-mm, no obvious differences in intensity were observed between the chicken breast fillets and the visceral bone fragments (Figure 6d). Thus, it is difficult to detect bone fragments using a single wavelength spectrum.

### 3.2. Clustering of Embedded Bone Fragments Using PCA

Table 2 shows the number of pixels (spectra) collected when the ROI for each part was extracted in a circular shape, as shown in Figure 6a. Therefore, the ROI pixels were collected from 5 exposed bone fragments, 5 embedded bone fragments, and 1 chicken breast meat for each of the 3 samples. The ROI pixels extracted from the exposed bone fragments were collected from a minimum of 16 pixels to a maximum of 36 pixels for each bone fragment thickness. The ROI extraction location of the embedded bone fragments under the chicken breast meat was roughly estimated and collected. The ROI range of chicken breast was extracted in a relatively wider region rather than the sum of the 5 exposed bone fragments, and a minimum 187 pixels to a maximum of 209 pixels were collected. 

**Figure 7:**
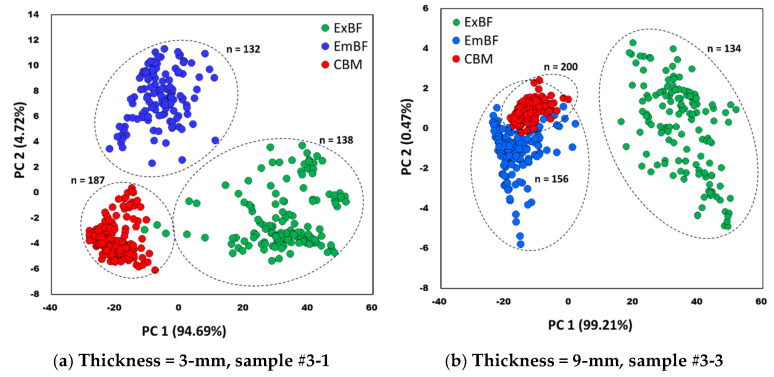
PCA score plots of the hyperspectral reflectance spectra of the 3 groups (ExBF, EmBF, and CBM).

Using the first and second PCs, the PCA model was developed for the classification of 3 groups—exposed bone fragments (ExBF), embedded bone fragments (EmBF), and chicken breast meat (CBM). The PCA score plot of these 3 groups is presented in Figure 7. Figure 7a shows a score plot for sample 3-1 with a fillet thickness of 3-mm, and Figure 7b shows a score plot for sample 3-3 with a fillet thickness of 9-mm. Regardless of the fillet thickness, the reflective spectra extracted from CBM were denser than the reflective spectra extracted from ExBF or EmBF, forming clusters (red dots). As shown in Figure 7a, the 3 groups of CBM, ExBF, and EmBF did not overlap with each other and were well grouped in the 3-mm thick sample. However, as shown in Figure 7b, when the thickness was 9-mm, it was difficult to distinguish the EmBF and CBM on the left side based on 0 of PC1. Although it was possible to visually cluster the sample groups using the PCA score plot, it was judged that there was a limit to distinguishing the differences between similar groups. 

### 3.3. Detection of Embedded Bone Fragment Using Subtraction Image

**Figure 8:**
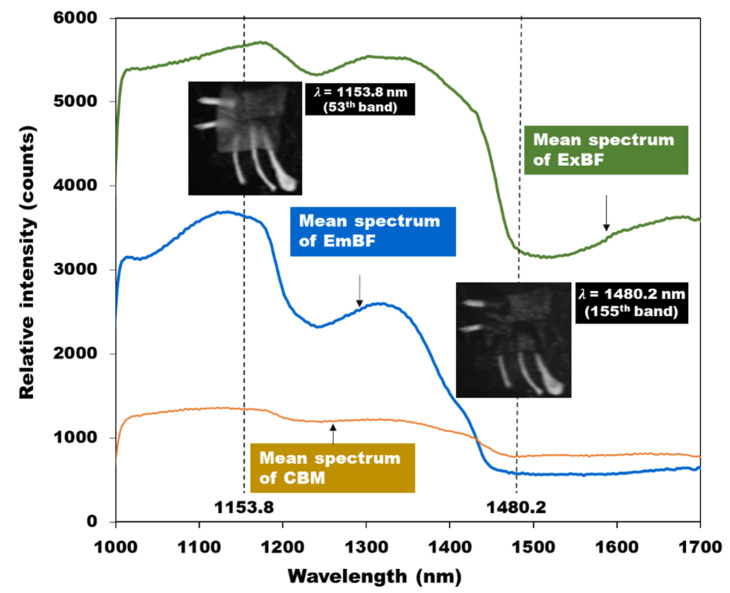
Comparison of mean spectra of ExBF, EmBF, and CBM in chicken breast fillets.

Two wavelength bands were selected to detect bone fragments embedded in the chicken breast fillets. The subtraction image was produced using the wavelength representing the highest and the lowest reflection intensity in the region of the bone fragment. Based on the spectral curves in Figure 8, the two wavelength bands of 1153.8-nm (53rd band) and 1480.2-nm (155th band) could be potential candidates for discriminating EmBF in chicken breast fillets. 

**Figure 9:**
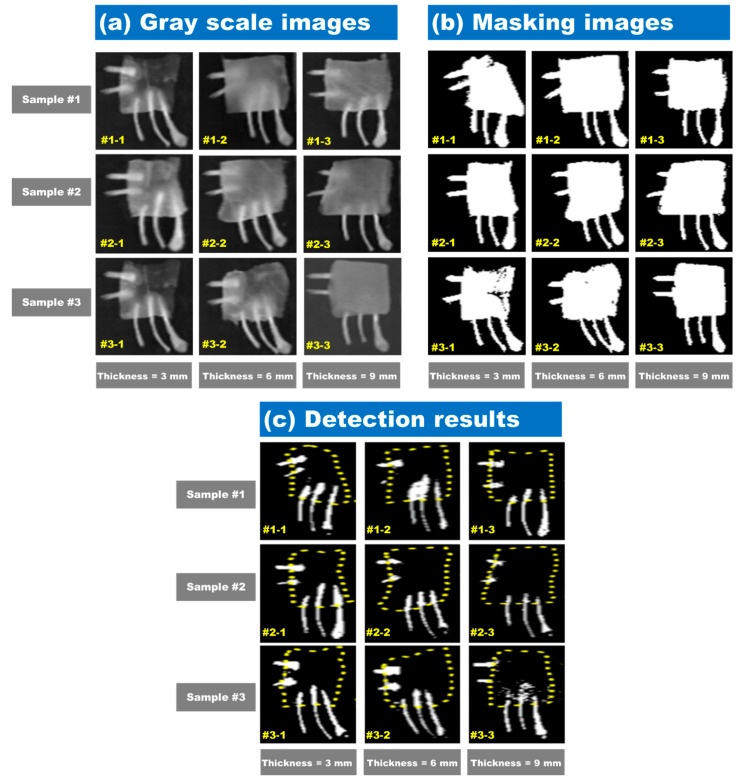
Hyperspectral images of chicken breast fillet samples obtained after applying the subtraction algorithm (${\mathrm{HSI}}_{1153.8}-{\;\mathrm{HSI}}_{1480.2})$: (**a**) gray scale images, (**b**) binarization results based on the threshold intensity (2200), and (**c**) detection results of bone fragments in chicken breast fillets obtained after applying the subtraction image algorithm.

A subtraction image algorithm was used to identify the minimal change between images of EmBF and CBM. The pixel values of ROI were extracted from subtraction images formed by 1153.8-nm and 1480.2-nm band images. Next, the threshold value for distinguishing the bone fragment was determined to be 2000, and this threshold was applied to the subtraction image model. Figure 9 shows the result of confirming the detection of foreign materials by applying the subtraction image model obtained above to all pixels of the hyperspectral image. Figure 9a shows a gray scale image with a subtraction algorithm. The binary masks of the chicken breast fillets are illustrated in Figure 9b. For removing the background, a threshold of (2200) was applied, and we multiplied the binary image with the subtraction image. Figure 9c shows the final result for the detection of bone fragments and chicken breast fillets by checking the threshold of the subtraction image algorithm. The yellow dashed line indicates the chicken breast fillet position. As shown in Figure 9c, for sample 1 with a thickness of 6-mm, No. 2 and No. 3 EmBF were easily detected against the No 4 sample. In addition, in the case of sample 3 with a thickness of 9-mm, the exposed areas of the No 4 and No 5 samples of the bone fragments were clearly visible, whereas the detection of the EmBF was not perfect. It was validated that the bone fragments were mostly detected, and the success score of detecting bone fragments using the subtraction image algorithm was 93.3%.

## 4. Conclusions and Outlook 

The purpose of this study was to examine the possibility of using near infrared hyperspectral imaging for detecting foreign substances that frequently occur in the meat processing industry. The meat used was chicken breasts, and 5 pieces of bone were used as the foreign material that were directly obtained by workers at the industrial site. 

A reflective spectrum was obtained for nine chicken breast fillets. The acquired spectra are pixel spectra and mean spectra. The measured data are dark reference, white reference, and spectral images of the sample. From the measured spectral data, hyperspectral image data were corrected using the dark reference and white reference. Then, the mean and pixel spectra of the samples were extracted from the calibrated hyperspectral image data. Distinctions between the ExBF and EmBF were confirmed using PCA. The results of PCA analysis showed that the 3-mm thick chicken breast pellet sample could be distinguished without any overlap, but some parts overlapped for the 9-mm thick sample. Wavelengths of 1153.8-nm (53rd band, strong reflection intensity in the average spectrum of bone fragments embedded in the chicken breast fillets) and 1480.2-nm (155th band, weak reflection intensity in the average spectrum of ExBF) were selected from the hyperspectral image. The subtraction image was obtained using the difference between the two wavelengths. After multiplying the subtraction image with the background removing image, which was extracted using a threshold value of 2200, a final binary image was obtained for detection of bone fragments. Among the 45 bone fragments embedded in all nine samples, 42 bone fragments were detected, resulting in a detection accuracy of 93.3%. In our case, 3 bone fragments were not detected. 

Future research will need to focus on improving the detection accuracy of the algorithm. In conclusion, our findings show that NIR hyperspectral reflective imaging techniques can be used to detect bone fragments in chicken breast. Such imaging techniques have many interesting perspectives from the qualitative and quantitative aspects, especially in the meat industry. The non-destructive characteristics multivariate of this technique provide an innovative platform to assess meat quality in laboratories and in the processing field. In future studies, the algorithm should be applied to detect foreign substances in other meats such as beef and pork. Considering these limitations are overcome, this technique can be applied for food processing and measurement of quality. 

## Figures and Tables

**Table 1:** Specifications of the SWIR hyperspectral imaging system.

Parameters	Values
Spectral range	987–1701 nm
Spatial dimensions	638 × 150 pixels
Exposure time	20 ms
Spectral increment	3.34 nm
Spectral resolution	6 nm
Image resolution	0.575 mm pixel^−1^
Moving speed of the translation stage	~46 mm s^−1^
Frame rate	98 fps

**Table 2:** Number of ROI pixels collected from 5 exposed bone fragments, 5 embedded bone fragments, and 1 chicken breast meat for each sample.

Meat Thickness	Number of Pixels Obtained from Each ROI														
	Exposed Bone Fragments (ExBF)							Embedded Bone Fragments (EmBF)							Chicken Breast
	1	2	3	4	5	Sum		1	2	3	4	5	Sum		Meat (CBM)
***Sample #1***															
3-mm (#1-1)	36	18	30	30	35	149		36	32	40	35	40	183		189
6-mm (#1-2)	32	16	32	16	28	124		30	28	28	35	28	149		204
9-mm (#1-3)	36	26	21	30	28	141		36	40	32	35	35	178		189
***Sample #2***															
3-mm (#2-1)	36	39	32	24	30	161		32	24	21	30	30	137		210
6-mm (#2-2)	28	24	30	28	28	138		40	40	24	30	30	164		198
9-mm (#2-3)	32	30	24	28	36	150		36	36	32	28	36	168		209
***Sample #3***															
3-mm (#3-1)	33	36	24	21	24	138		32	24	20	28	28	132		187
6-mm (#3-2)	30	33	20	16	24	123		24	28	24	30	32	138		208
9-mm (#3-3)	28	30	30	22	24	134		32	36	32	28	28	156		200
